# Trendy I Oczekiwania w Badaniach Podłoża Molekularnego Encefalopatii Padaczkowych - Stan Na Rok 2017

**DOI:** 10.34763/devperiodmed.20172104.317327

**Published:** 2018-01-02

**Authors:** Dorota Hoffman-Zacharska, Paulina Górka-Skoczylas

**Affiliations:** 1 Zakład Genetyki Medycznej, Instytut Matki i Dziecka, Warszawa, Polska

**Keywords:** padaczka, encefalopatia padaczkowa, heterogenność genetyczna diagnostyka molekularna, epilepsy, epileptic encephalopathy, genetic heterogeneity, molecular diagnostics

## Abstract

Padaczka jest częstą chorobą neurologiczną, diagnozowaną u 0,8-1% populacji światowej. Jest to choroba głównie wieku rozwojowego, gdyż 80% zachorowań dotyczy osób poniżej 20 r.ż. Przyczyny padaczki są zróżnicowane, jednak zawsze uważana była za chorobę o podłożu genetycznym, do czego w chwili obecnej nie mamy już wątpliwości. Genetyka padaczek przeszła rewolucję od momentu identyfikacji pierwszego „genu padaczkowego”. Stało się tak za sprawą wprowadzenia sekwencjonowania następnej generacji jako podstawowej metody badawczej i diagnostycznej, oraz zmiany podejścia badawczego, z analizy padaczek rodzinnych do badań podłoża, głównie sporadycznych, encefalopatii padaczkowych. W krótkim czasie w oparciu o identyfikacje genów sprawczych wyodrębniono ponad 50 zespołów wczesnodziecięcych encefalopatii padaczkowych. Obecnie sekwencjonowanie następnej generacji, eksomowe lub panelowe wykorzystywane jako powszechne narzędzie w diagnostyce encefalopatii padaczkowych o skuteczności diagnostycznej na poziomie 30-40%. Uzyskanie „diagnozy genetycznej” w coraz większej liczbie przypadków pozwala na zastosowanie odpowiedniej dla danego przypadku farmakoterapii. Jako że encefalopatie padaczkowe uznawane są za choroby modelowe dla padaczek, opracowywane dla nich strategie terapeutyczne mogą przyczynić się także do opracowania leczenia częstych postaci tej choroby.

## Abstract (en)

Epilepsy is common neurological condition affecting 0.8-1% of the human population. Since 80% of patients are under 20 years of age, it is mainly a disease of the developmental period. The causes of epilepsy are heterogeneous, but the disease has always been considered a genetic disorder, which no longer doubted. Epilepsy genetics has undergone a revolution since the discovery of the first gene responsible for epilepsy. This is mainly because of introduction of the next generation sequencing as research and diagnostic tool, and transition from studies of pedigrees with epilepsy to the analysis of cases of epileptic encephalopathies. In a short time more than 50 early infantile epileptic encephalopathies were recognized due to the causative genes. Whole exome or targeted panel sequencing has been used as a diagnostic tool with a diagnostic yield of about 30-40%. The „genetic diagnosis” that is obtained makes it possible to introduce targeted treatment in an increasing number of cases. Since epileptic encephalopaties are often regarded as the model disease for epilepsy, these therapeutic strategies can provide treatment for patients with common epilepsies.

Padaczka to choroba, która towarzyszy człowiekowi od zawsze, a pierwsze o niej wzmianki znajdziemy już w dokumentach babilońskich. To jedna z najczęstszych chorób neurologicznych dotykająca 0,8-1% wszystkich populacji. Według danych Światowej Organizacji Zdrowia (*ang*. World Health Organisation, WHO), na świecie na padaczkę choruje ponad 50 milionów osób, a liczbę nowych zachorowań określa się na 2,4 miliona/rok. W Europie liczba chorych szacowana jest na około 3,6 miliona, w Polsce to około 350 tysięcy osób. Padaczka jest przede wszystkim chorobą wieku rozwojowego, bo aż 80% zachorowań stwierdza się poniżej dwudziestego roku życia. W pierwszym roku życia odnotowuje się najwyższą częstość zachorowania na ciężkie, lekooporne zespoły padaczkowe o złym rokowaniu dla pacjentów – encefalopatie padaczkowe (*ang*. Epileptic Encephalopathies, EEs). W tej też grupie wiekowej najważniejszą rolę w etiologii tych chorób odgrywają czynniki genetyczne.

Pomimo, że padaczka towarzyszy człowiekowi od tysięcy lat to tak naprawdę zaczęto ją dokładniej poznawać i rozumieć dopiero w ostatnich wiekach [1]. Najpierw jako zaburzenie pobudliwości komórek nerwowych mózgu (rozwój technik EEG w wieku XX), a następnie poznając jej podłoże na poziomie molekularnym i komórkowym poprzez identyfikację genów, których mutacje prowadzą do rozwoju padaczki (rozwój technik mapowania genów oraz sekwencjonowania następnej generacji na przełomie XX/XXI). Jak wskazują najnowsze rekomendacje Międzynarodowej Ligi Przeciwpadaczkowej (*ang*. International League Against Epilepsy; ILAE) dotyczące klasyfikacji padaczek, ostateczna diagnoza uwzględniać musi nie tylko rodzaj napadów czy typ padaczki, ale także, w równym stopniu jej etiologię - w tym podłoże genetyczne choroby [2].

W XIX w. John Russell Reynolds w swej pracy „*Epilepsy: Its Symptoms, Treatment and Relation to Other Chronic Convulsive Diseases*” pisał „*Poczynając od prac starożytnych, autorzy byli i są nadal zgodni, co do teorii, że padaczka jest przede wszystkim chorobą dziedziczną…*” – nie potrafiono jednak tego udowodnić. Dzisiaj podłoże genetyczne padaczek/zespołów padaczkowych nie podlega już dyskusji. Badania genetyczne stały się również elementem różnicowej diagnostyki zespołów padaczkowych. Szczególnie dotyczy to zespołów z grupy encefalopatii padaczkowych. identyfikacja podłoża molekularnego pozwala tu na jednoznaczną weryfikację rozpoznania, a co najważniejsze, w coraz większej liczbie przypadków możliwość zastosowania odpowiedniego, dla zidentyfikowanego defektu molekularnego, leczenia czy postępowania terapeutycznego. Tak więc odnosząc się do dyskusji rozpoczętej identyfikacją pierwszych „*genów padaczkowych*”, a której wyrazem była debata na łamach czasopisma Epilepsia „*Does genetic information in humans help us treat patients?”* [3] zdecydowanie z roku na rok argumentów popierających tezę, że „tak” jest coraz więcej, co wynika głównie z nowych danych na temat etiopatogenezy poszczególnych zespołów. Przyczynia się do tego wprowadzanie do diagnostyki nowych, bardziej skutecznych technik molekularnych np. sekwencjonowania następnej generacji (*ang*. Next Generation Sequencing, NGS).

NGS to bardzo wydajne narzędzie pozwalające na równoczesną analizę fragmentów DNA pokrywających cały genom – sekwencjonowanie całogenomowe (ang. Whole Genome Sequencing; WGS), tylko fragmenty kodujące genomu (eksom) – sekwencjonowanie eksomowe (*ang*. Whole Exom Sequencing, WES), albo wybrane geny/obszary genomu – sekwencjonowanie panelowe (celowane). Każda z wymienionych powyżej metod identyfikuje szereg wariantów DNA, wśród których trzeba zidentyfikować te, które konkretnie odpowiedzialne są za fenotyp badanego. Nie zawsze jest to możliwe, nie zawsze również potrafimy jednoznacznie określić patogenność wytypowanych wariantów, nawet jeżeli zlokalizowane są w znanych i wiązanych z daną jednostką chorobową genach.

Badania genetyczne zawsze obciążone były i nadal są problemem niepewności (ang. uncetainty), zarówno w procesie diagnostycznym, co do identyfikacji przyczyny choroby (heterogenność obrazu klinicznego, heterogenność genetyczna chorób), jak i w zakresie poradnictwa genetycznego (mutacje de novo, mozaikowość, niepełna penetracja mutacji czy zmienna ekspresywność). Medycyna genomowa, poprzez liczbę uzyskiwanych w badaniach danych, nie zawsze możliwych do interpretacji, zdecydowanie zwiększa ten zakres niepewności. Dlatego już na etapie kierowania pacjenta na badanie i w procesie poradnictwa genetycznego problem ten powinien być wyraźnie akcentowany. Szczególnie że w ostatnich latach popularny stał się przekaz, że poznanie sekwencji genomu lub wszystkich genów pacjenta/badanego na pewno pozwoli na zidentyfikowanie przyczyn jego choroby [4]. Należy także zwrócić uwagę na fakt, że molekularne badania diagnostyczne, w tym NGS, znajdują zastosowanie w stosunku do chorób rzadkich, w większości monogenowych. Musimy więc zdawać sobie sprawę, że odnosimy się tutaj do niewielkiej grupy padaczek/zespołów padaczkowych. Obecnie szacuje się, że choroby monogenowe, których padaczka jest głównym lub jedynym objawem stanowią niewielki odsetek zespołów padaczkowych (~10%), natomiast w przypadku większości jednostek ich dziedziczenie jest najprawdopodobniej poligenowe lub wieloczynnikowe.

## Padaczki – Badania genetyczne i molekularne

Pierwszym etapem rozwoju genetyki padaczek było wykazanie udziału czynników genetycznych w rozwoju tej choroby, a pierwszym krokiem badania rodzinne i badania bliźniąt prowadzone na początku XX w. Wykazały one istotność udziału czynników dziedzicznych w rozwoju padaczki, lecz były niedopracowane metodologicznie, między innymi pod kątem kryteriów włączenia w odniesieniu do etiologii choroby. Istotną rolę odegrały dopiero badania prowadzone w latach 50-tych przez Lennox’a, w których udowodnił rodzinne predyspozycje występowania padaczki. W badaniach bliźniąt wykazał ponadto, że ryzyko wystąpienia padaczki „idiopatycznej” u bliskich krewnych pacjenta jest wyższe niż padaczki symptomatycznej [5]. Badania przeprowadzone przez Berkovic’a w latach dziewięćdziesiątych XX w. potwierdziły wyniki Lennox’a, również w aspekcie udziału czynników genetycznych jak i środowiskowych w etiologii choroby (w różnym stopniu, zależnie od rodzaju padaczki). Analiza objęła 253 par, gdzie u jednego lub obojga z bliźniąt stwierdzano napady. W badanej grupie wykazano zgodność pod względem napadów dla bliźniąt monozygotycznych (MZ) na poziomie 0.62 natomiast dla bliźniąt dizygotycznych (DZ) 0.18. Stwierdzono również, większą zgodność diagnozowanego wśród bliźniąt MZ zespołu (MZ vs. DZ, 94% vs. 74%), co wskazuje na wpływ czynników genetycznych raczej na typ padaczki, a nie ogólną predyspozycje do wystąpienia napadów [6]. Co ciekawe przeprowadzono w tym samym czasie reanalizę danych badań Lennox’a – dla 75% opisanych chorych ustalono rodzaj zespołu wg. obowiązującej klasyfikacji ILAE [7], i wykazano, jak i w późniejszych badaniach Brekovic’a, specyficzność czynników genetycznych w odniesieniu do zespołu padaczkowego występującego u bliźniąt (MZ vs. DZ, 86% vs. 60%) [8]. Udział czynników genetycznych w rozwoju padaczek/zespołów padaczkowych przyjmuje się obecnie na poziomie 40-60%.

Padaczki, podobnie jak i wiele innych chorób o podłożu genetycznym możemy podzielić na tzw. częste (*ang*. common) lub rzadkie, monogenowe (*ang*, rare, monogenic). Określenie padaczki częste jest pojęciem ogólnym i obejmuje jednostki, które występują u ~1 na 200 osób z nawracającymi napadani uogólnionymi lub częściowymi [9]. Częste padaczki/zespoły padaczkowe mają podłoże wieloczynnikowe, a ich fenotyp jest wynikiem interakcji szeregu współdziałających genów oraz ich zmienności. Grupa ta obejmuje szereg najczęściej diagnozowanych zespołów padaczkowych: genetyczne padaczki uogólnione (*ang*. Genetic Generalized Epilepsies, GGE) w tym młodzieńczą padaczkę miokloniczną (*ang*. Juvenile Myoclonic Epilepsy, JME) i idiopatyczną padaczkę z napadami nieświadomości (*ang*. Idiopathic Absence Epilepsy, IAE), a także padaczki ogniskowe (*ang*. Focal Epilepsy, FE) obejmujące padaczkę skroniową (*ang*. Temporal Lobe Epilepsy, TLE) i Idiopatyczną padaczkę ogniskową (*ang*. Idiopathic Focal Epilepsy, IFE). W przypadku tej grupy chorób nie można jednoznacznie określić „*czynnika genetycznego/genu*” odpowiedzialnego za wystąpienie choroby. W genomowych badaniach asocjacyjnych (*ang*. Genome-Wide Association Study, GWAS), przeprowadzonych dla odpowiednio licznych kohort pacjentów zidentyfikowano szereg *loci* i polimorfizmów powiązanych z tą grupą padaczek oraz wykazano, że powszechne/częste polimorfizmy jednego nukleotydu (*ang*. common Single Nucleotide Polymorphisms, cSNPs) w naszym genomie odpowiadają za około 26% zmienności fenotypowej padaczek. Szacunki z tych badań wskazują na zaangażowanie co najmniej 400 *loci* związanych z podatnością do wystąpienia padaczek, aczkolwiek może ich być nawet kilka tysięcy − każde o małym czynniku ryzyka [10]. Chorym i rodzinom chorych z padaczkami z tej grupy (GGE występuje u 30-40% chorych z padaczką) nie można obecnie zaproponować diagnostycznego testu genetycznego, a dane o ryzyku zachorowania dla krewnych chorego opiera się na wynikach badań populacyjnych. Dla krewnych pierwszego stopnia chorych z GGE wynosi ono 8-12%, podczas gdy dla krewnych drugiego stopnia równe jest z ryzykiem populacyjnym (1-2%).

Zespoły padaczkowe rzadkie − monogenowe (o dziedziczeniu mendlowskim) to choroby, w przypadku których mutacja pojedynczego genu jest niezbędna i wystarczająca dla wystąpienia określonego fenotypu, oraz związana z jego segregacją w rodzinie. Pomimo, że zespoły te stanowią niewielki odsetek w grupie padaczek to identyfikacja ich genów sprawczych pozwala następnie na identyfikację zarówno szlaków molekularnych jak i komórkowych zaangażowanych w ich etiopatogenezę.

Początek genetyki padaczek monogenowych to rok 1994, gdy zidentyfikowano pierwszy gen *- CHRNA4*, którego mutacje stanowiły podłoże nocnej padaczki czołowej o dziedziczeniu autosomalnym dominującym (*ang*. Autosomal Dominant Nocturnal Fronatl Lobe Epilepsy, ADNFLE). Przez następne kilka lat badając rodzinne przypadki różnych rodzajów epilepsji/zespołów padaczkowych z zastosowaniem analizy sprzężeń zidentyfikowano kilkanaście genów, których mutacje stanowiły podłoże molekularne choroby. Kodowane przez nie białka w większości stanowiły kanały/podjednostki kanałów jonowych zależnych od napięcia lub ligandu. Wtedy też powstało pojęcie padaczek jako „*kanałopatii”* (*ang*. channelopathies).

Jednym ze zidentyfikowanych w tym okresie genów, był kodujący białko Nav1.1 - podjednostkę α napięciowozależnego kanału sodowego, gen *SCN1A*. Wykazano, że jest związany z występowaniem zespołu uogólnionej padaczki z drgawkami gorączkowymi plus (*ang*. Generalized Epilesy with Febrile Seizures Plus, GEFS+; według obecnej nomenklatury zespół genetycznie uwarunkowanej padaczki z drgawkami gorączkowymi plus, *ang*. Genetic Epilesy with Febrile Seizures Plus, GEFS+) o dziedziczeniu dominującym i zmiennym obrazie fenotypowym [11]. W 2001 roku z mutacjami w tym samym genie powiązano występowanie ciężkiej encefalopatii padaczkowej – zespołu Dravet (*ang*. Dravet Syndrome, DS). W tym wypadku były to jednak głównie mutacje o charakterze *de novo* [12]. Wykazano wtedy po raz pierwszy występowanie szerokiego spektrum fenotypowego padaczek – od zespołów stosunkowo łagodnych klinicznie do ciężkich, lekoopornych o złym rokowaniu, powodowanych mutacjami w jednym genie (ryc. 1). Z drugiej strony analiza podłoża genetycznego w rodzinach z GEFS+ ujawniła problem heterogenności genetycznej tego zespołu, co okazało się później powszechne w odniesieniu także do innych zespołów padaczkowych (tab. I).

**Tabela I:** Zróżnicowanie fenotypowe zespołów padaczkowych o takim samym podłożu molekularnym – mutacjami w tym samym genie, wg. Helbig i wsp. 2016 [14]. Table I. Epileptic syndrome heterogeneity having the same molecular background – mutations in one gene, according to Helbig et al. 2016 [14].

Fenotyp łagodny *Mild phenotype*	Nazwa genu *Gene*	Fenotyp ciężki *Severe phenotype*
Genetyczna padaczka z drgawkami gorączkowymi plus (GEFS+3) *Genetic epilepsy with febrile seizures plus (GEFS+3)*	*SCN1A*	Wczesnodziecięca encefalopatia padaczkowa – zespól Dravet *Early infanle epilepc encephalopathy type 6 (EIEE6)*
Łagodna rodzinna padaczka niemowląt *Benign familial neonatal epilepsy (BFNE)*	*SCN2A*	Noworodkowa encefalia padaczkowa *Neonatal epileptic encephalopathy – (EIEE11*)
Łagodna rodzinna padaczka niemowląt *Benign familial neonatal epilepsy (BFNE)*	*SCN8A*	Wczesnodziecięca encefalopatia padaczkowa *Early infantile epileptic encephalopathy type* 6 (EIEE13)
Łagodne rodzinne drgawki okresu noworodkowego *Benign familial neonatal seizures (BFNS)*	*KCNQ2*	Noworodkowa encefalia padaczkowa *Neonatal epileptic encephalopathy – (EIEE7*)
Rodzinna padaczka ogniskowa *Familial focal epilepsy*	*SLC2A1*	Noworodkowa encefalia padaczkowa – zespół niedoboru transportera glukozy GLUT1 *GLUT1 Deficiency Syndrome – (GLUT1-DS1)*
Młodzieńcza padaczka miokloniczna *Juvenile myoclonic epilepsy*	*GABRA1*	Noworodkowa encefalia padaczkowa *Neonatal epileptic encephalopathy – (EIEE19)*
Genetyczna padaczka z drgawkami gorączkowymi plus (GEFS+3 ) *Genetic epilepsy with febrile seizures plus (GEFS+3)*	*GABRG2*	Wczesnodziecięca encefalopatia padaczkowa *Early infantile epileptic encephalo*pathy
Zespoły padaczki z afazją	*GRIN2A*	Noworodkowa encefalia padaczkowa *Neonatal epileptic encephalopathy*
Łagodna rodzinna padaczka niemowląt *Benign familial neonatal epilepsy (BFNE)*	*PRRT2*	Noworodkowa encefalia padaczkowa Neonatal epileptic encephalopathy

Przełomem w badaniach nad podłożem molekularnym padaczek, związanym z rozwojem molekularnych technik badawczych, był rozwój genomiki i przejście z analizy poszczególnych genów do analizy na poziomie genomowym. Patrząc z perspektywy roku 2017 można wyróżnić kilka etapów rozwoju badań na padaczkami. Są to, jak określają autorzy koncepcji: 1. „*era kanałopatii*” (*ang*. „*channelopathy era*”) okres, do 2001 roku, identyfikacji niewielkiej liczby genów kodujących głównie kanały jonowe (np. *CHRNA4, SCN 1B, KCNQ2 i 3, SCN1A, GABRG2)*, 2. „*mroczne czasy*” (ang. „*dark ages*”) genetyki padaczek, kiedy prowadzono szereg badań asocjacyjnych, bez konkluzywnych i powtarzalnych wyników, oraz 3. „*era genomiki”* rozpoczęta okresem badań z zastosowaniem porównawczej hybrydyzacji genomowej do mikromacierzy (*ang*. array comparative genomic hybridization, aCGH) a następnie zdominowana przez metodę NGS – okres „*bańki NGS*” (ang. „*NGS buble*”), który trwa do dzisiaj [13, 14]. W badaniach z zastosowaniem aCGH i NGS istotna była też zmiana podejścia badawczego, w centrum uwagi znalazły się nie padaczki rodzinne, ale bardzo specyficzna grupa zespołów padaczkowych – encefalopatie padaczkowe, dlatego też okres badań z zastosowaniem techniki NGS nazywa się także „*erą encefalopatii padaczkowych*”.

**Ryc. 1:**
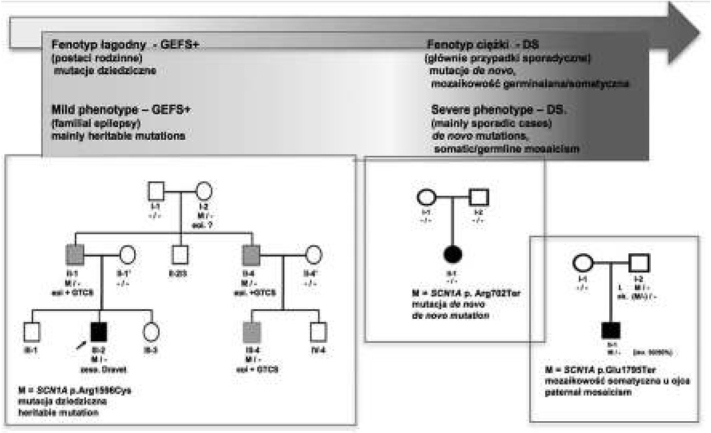
Spektrum zespołów padaczkowych związanych z mutacjami w genie *SCN1A*; od fenotypu łagodnych w przebiegu, ale heterogennych pod względem obrazu klinicznego rodzinnych zespołów GEFS+ do ciężkiej encefalopatii padaczkowej – zespołu Dravet, pojawiającego się zazwyczaj sporadycznie w wyniku mutacji de novo. DS u rodzeństwa jest wynikiem mozaikowości dla mutacji w genie *SCN1A* występującej u jednego z rodziców. Fig. 1. The spectrum of SCN1A- related epilepsy syndromes; from generalized epilepsy with febrile seizures plus (GEFS+) at the mild end to Dravet syndrome − severe epileptic encephalopathy at the severe end. GEFS+ is a heritable form of epilepsy characterized by phenotype heterogeneity, DS is rather sporadic due to de novo mutations. DS reported in siblings results from parental mosaicism in the SCN1A gene.

Analiza mikrorearanżacji z zastosowaniem aCGH pozwoliła na zidentyfikowanie nawracających mikrodelecji 15q13.3, 15q11.2, and 16p13.11, identyfikowanych u pacjentów z GGEs z częstością około 1% każda. Późniejsze badania wykazał, że zmiany liczby kopii (*ang*. Copy Number Variations, CNVs) odgrywają istotną rolę u osób z padaczką. Obecnie szacuje się, że około 4% chorych z encefalopatiami padaczkowymi ma patogenne CNVs, a u kolejnych 4% identyfikowane są warianty potencjalnie patogenne [15, 16]. Systematyczna analiza delecji i duplikacji u pacjentów z padaczkami o ciężkim przebiegu pozwoliła też na identyfikację konkretnych genów stanowiących ich podłoże jak np. *STXBP1*, u pacjenta z zespołem Ohtahara [17] czy *GRIN2A* u pacjentów z EEs [18]. Obecnie gen *STXBP1* jest jednym z najczęściej identyfikowanych genów u osób z EEs o szerokim spektrum fenotypowym, a *GRIN2A* u chorych z zespołem padaczki z afazją. Warto wspomnieć tu również o genie *CHD2*, który został zidentyfikowany przez analizę mikrodelecji u pacjenta z wczesnodziecięcą encefalopatia padaczkową (*ang*. Early Infantile Epileptic Encephalopathy, EIEE), ale także w ramach pierwszych kompleksowych, wieloośrodkowych badaniach eksomowych z zastosowaniem NGS [19].

Na początku 2017 r. Wang i wsp. [20] przeprowadzili analizę danych dostępnych w szeregu bazach dotyczących genów powiązanych z występowaniem padaczki. Wyodrębnili 997 genów, które zakwalifikowano do czterech grup zależnie od udziału padaczki w fenotypie: (1) 84 „*geny padaczkowe*” związane z padaczką/zespołami padaczkowymi, gdzie jest ona podstawowym objawem choroby, (2) 73 „*geny neurorozwojowe*” związane z dużymi wadami rozwojowymi mózgu i padaczką, (3) 536 „*geny związane z padaczką”* czyli powodujące jednostki chorobowe, w których padaczka stanowi jeden z objawów, (4) 284 *„geny potencjalnie związane z padaczką”*, których udział w patogenezie choroby wymaga weryfikacji [20]. Wśród tych genów znajduje się ponad 70 wiązanych z występowaniem encefalopatii padaczkowych, których analiza powala na identyfikacje około 20-25% ciężkich padaczek o wczesnym wieku zachorowania [14].

## Encefalopatie padaczkowe - Definicja zespołu

Encefalopatie padaczkowe traktowane są jako zespoły, w których objawem wiodącym jest padaczka, stanowiąca pierwszy cel terapeutyczny, a głównym objawem współwystępującym jest zahamowanie lub regres rozwoju. Stanowią one bardzo specyficzną, choć heterogenną, grupę rzadkich zespołów padaczkowych ujawniających się we wczesnym okresie rozwojowym. W przypadku EEs objawy zazwyczaj występują już w niemowlęctwie, a 40% padaczek występujących przed 3 r.ż. należy do tej grupy. EIEEs cechuje współwystępowanie określonego obrazu klinicznego, charakterystycznego zapisu EEG oraz nieprawidłowy rozwój psychoruchowy pacjentów. Są to ciężkie, lekooporne zespoły padaczkowe o złym rokowaniu, w których, występowanie napadów i/lub podklinicznych wyładowań epileptogennych, jak się przyjmuje, stanowi podłoże postępujących zaburzeń funkcjonalnych mózgu prowadząc do zahamowania (niekiedy regresu) rozwoju pacjenta [21]. Obecnie taka definicja EIEEs nie jest już przyjmowana bez zastrzeżeń. Zwraca się uwagę na fakt, że w przypadku niektórych zespołów oba fenotypy rozwijają się niezależnie. Tak jest w przypadku encefalopatii powodowanej mutacjami w genie *CDKL5*, gdzie u większości pacjentów najpierw występuje opóźnienie rozwojowe [22]. Natomiast u pacjentów z zespołem Dravet i mutacjami w genie *SCN 1A* do zahamowania rozwoju często dochodzi w drugim roku życia i wydaje się, że bez bezpośredniego wpływu napadów padaczkowych [23]. Możliwe są więc dwa scenariusze, gdzie czynniki genetyczne prowadzą do niezależnego rozwoju obu fenotypów lub gdzie padaczka jest czynnikiem powodującym zaburzenia neurorozwojowe. Biorąc je pod uwagę zaproponowano podział EIEEs na dwie grupy: 1. „*prawdziwe*” EEs w przypadku, których u początkowo prawidłowo rozwijających się dzieci aktywność padaczkowa prowadzi do zaburzeń neurorozwojowych; przykładem są tu zespoły ciągłych wyładowań iglica-fala podczas snu wolnofalowego (*ang*. Continuous Spikes and Waves during sleep Syndrome, CSWS) czy zespól Landau-Kle+nera (*ang*. Landau-Kle+ner Syndrome, LKS), 2. grupa obejmująca choroby, w przypadku których zaburzenia kognitywne są obserwowane już w momencie wystąpienia napadów i są wynikiem zaburzeń formowania sieci neuronalnej. Bioelektryczna aktywność napadowa mózgu w tym może stanowić tylko czynnik pogarszający. Ta grupa obejmuje zespoły o charakterystycznym wieku zachorowania i charakterystyce kliniczno-elektro-encefalograficznej; przykładem są tu zespół Ohtahara (*ang*. Ohtahara Syndrome), wczesna miokloniczna encefalopatia (*ang*. Early Myoclonic Encephalopathy, EME) oraz niektóre przypadki zespołów Westa, Dravet czy Lennox’a-Gastaut (*ang*. West, Dravet, Lennox-Gatsaut Syndromes; WS, DS., LGS) [24]. Biorąc pod uwagę fakt, że w przypadku tej grupy zespołów nie wszyscy pacjenci spełniają kryteria ich charakterystyki, oraz wzrastającą liczbę identyfikowanych genów, których mutacje powodują wystąpienie wczesnodziecięcych encefalopatii padaczkowych proponuje się określanie poszczególnych zespołów jako zależnych od mutacji w konkretnym genie (np. *SCN8A* zależna encefalopatia, EIEE13 wg. OMIM). Na pewno jest to propozycja warta rozważenia, szczególnie jeżeli weźmiemy pod uwagę heterogenność genetyczną i fenotypową tych zespołów (ryc. 2).

## Encefalopatie padaczkowe – Wyzwanie dla genetyka

Niepełnosprawność intelektualna (NI), to ciężkie zaburzenie neurorozwojowymi zdecydowanie redukujące płodność osób chorych, a mimo to występujące stosunkowo często w populacji ludzkiej (1-2%). Próbą wytłumaczenia tego zjawiska była hipoteza kompensacji utraty alleli w populacji poprzez wysoką częstość mutacji *de novo* w genomie człowieka [25]. EIEEs podobnie jak i NI w stopniu głębokim to choroby, które pojawiają się w rodzinach zazwyczaj sporadycznie i w większości przypadków są wynikiem mutacji germinalnych *de novo*. Udowodniły to pierwsze badania z zastosowaniem eksomowego sekwencjonowania następnej generacji – WES prowadzone dla pacjentów z NI [26, 27], które następnie w podobnym układzie eksperymentalnym zastosowano w wieloośrodkowych badaniach nad podłożem EIEEs [28]. W obu przypadkach do identyfikacji potencjalnie patogennych wariantów sekwencyjnych zastosowano podejście określane jako „*analiza trójek*” (*ang*. Trio analysis), tzn. porównanie sekwencji eksomów probanta i jego rodziców. Przy założeniu, że wariant patogenny jest dominujący i *de novo*, brano pod uwagę tylko te zmiany, które zidentyfikowane u probanta nie miały charakteru dziedzicznego. Dzięki takiemu podejściu badawczemu, w stosunkowo krótkim czasie zidentyfikowano szereg genów, których mutacje stanowią podłoże molekularne encefalopatii padaczkowych. W chwili obecnej w bazie OMIM wyodrębnionych jest 55 typów EIEEs przypisanych konkretnym genom, a lista na pewno nie jest jeszcze ciągle zamknięta [29].

**Ryc. 2:**
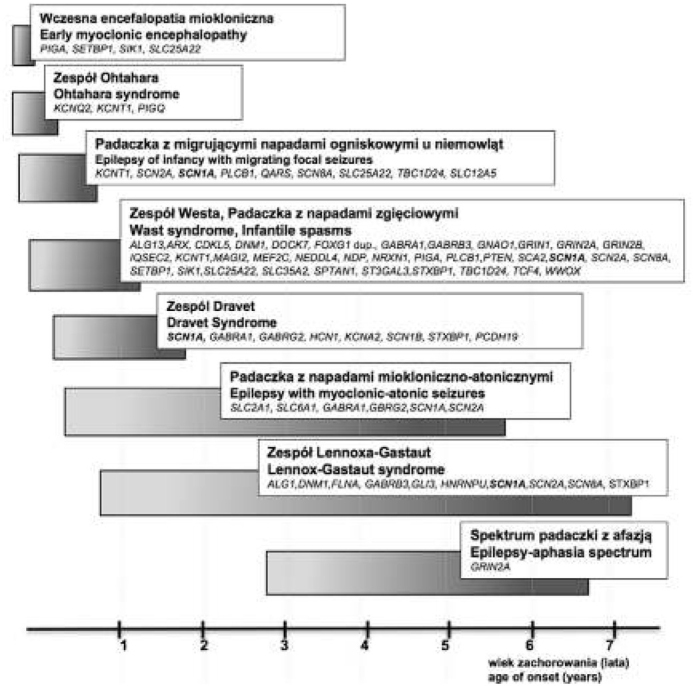
Heterogenność genetyczna i fenotypowa wczesnodziecięcych encefalopatii padaczkowych. Fig. 2. Genetic and phenotypic heterogeneity of early infantile epileptic encephalopathies.

Dużym problemem w badaniach genetycznych zespołów z grupy EIEEs pozostaje ich heterogenność genetyczna i kliniczna, ale także fakt ewolucji objawów klinicznych w czasie, co niejednokrotnie powoduje, że jednoznaczną diagnozę można postawić oceniając rozwój choroby retrospektywnie.

Jednym z najlepiej poznanych zespołów z tej grupy, zarówno pod względem obrazu klinicznego, jak i podłoża molekularnego, jest zespół Dravet (wczesnodziecięca encefalopatia padaczkowa typ 6, EIEE6) najczęściej powodowany mutacjami w genie *SCN 1A* (obecnie szacowana częstość populacyjna to 1:22 000). Mutacje w genie *SCN1A* są również przyczyną łagodniejszego, dziedzicznego zespołu GEFS+2 (Ryc. 1), gdzie obserwujemy często wewnątrzrodzinną zmienność obrazu klinicznego z wystąpieniem DS włącznie [11, 30].

Rozpoznanie kliniczne, czy podejrzenie DS jest wskazaniem do wykonania analizy genu *SCN1A*. Mutacje w genie *SCN1A* identfikowane są ponad 90% pacjentów z diagnozą DS, u pozostałych mogą to być mutacje *de novo* w genach *SCN2A, SCN1B, GABRA1, GABRG2, KCNA2, HCN1, PCDH19, CHD2, STXBP1* (Ryc.2) [31]. Najnowsze doniesienia Sadler i wsp., wskazują, że spektrum kliniczne chorób *SCN1A*-zależnych może być szersze niż przypuszczano, prezentując przypadki chorych z heterozygotyczną mutacją missens p.> r226Met o fenotypie wczesnej encefalopatii padaczkowej, ale o zdecydowanie cięższym przebiegu niż DS [32].

Dokładna analiza obrazu klinicznego dla większej grupy pacjentów ze zidentyfikowanymi mutacjami patogennymi w określonych genach pozwala na wyodrębnienie nowych jednostek chorobowych na podstawie cech różnicujących je od innych o podobnym fenotypie. Tak było również w przypadku zespołów związanych z mutacjami w genach *PCDH19, SCN8A i CHD2*. Zespoły te został wyłączone z grupy zespołów podobnych do DS (*ang*. Dravet Like Syndromes; DS-Like) i określone jako odrębne jednostki, odpowiednio jako EIEE9 i EIEE13 oraz dziecięca encefalopatia padaczkowa związana z genem *CHD2* (*ang. CHD2* related epileptic encephalopathy; *CHD2* EE) [33, 34, 19].

## Encefalopatie padaczkowe – Mechanizmy molekularne

Choroby monogenowe stanowią niewielki odsetek chorób genetycznie uwarunkowanych, ale to analiza ich podłoża – identyfikacja genu sprawczego, pozwala na poznanie mechanizmów i szlaków komórkowych zaangażowanych w ich etiopatogenezę. Badania identyfikujące geny, których mutacje związane są z ekspresją danego fenotypu, stanowią jednak dopiero pierwszy etap prowadzący do poznania patomechanizmów poszczególnych chorób. EIEEs to bardzo heterogenna grupa jednostek, których obraz kliniczny jest wynikiem określonych dysfunkcji układu nerwowego. Okres rozwojowy mózgu, a w szczególności ostatni okres rozwoju prenatalnego i pierwsze dwa lata rozwoju postnatalnego to tzw. „*okres krytyczny*” formowania równowagi pomiędzy procesami pobudzenia i hamowania neuronalnego OUN. Jest to okres, w którym charakterystyczne jest obniżenie progu pobudliwości związane z nierównowagą ekspresji np. kanałów jonowych pobudzających w stosunku do hamujących, skutkującą podwyższoną podatnością na wystąpienie napadów, które mogą prowadzić do zaburzenia procesów synaptogenezy i prawidłowego rozwoju sieci neuronalnych [35]. Wśród genów sprawczych EIEEs zidentfikowano do tej pory nie tylko kodujące kanały jonowe i receptory neurotransmiterów, ale także geny kodujące białka związane z przekazywaniem sygnału i stabilizacją synaps, regulacją ekspresji genów neuronalnych i rearanżacją chromatyny (Ryc. 3). Wskazuje to na udział wielu szlaków komórkowych w patogenezie EIEEs, których dysfunkcje mogą powodować zaburzenia interakcji neuronalnych, prowadząc do zachwiania homeostazy i metabolizmu komórkowego [36, 24, 20]. Zaangażowanie tak różnorodnych mechanizmów patogennych, przy niejednokrotnie niejednoznacznym obrazie klinicznym EiEEs stanowi poważny problem w doborze terapii. Tak więc poznanie podłoża choroby, ale także zrozumienie efektów mutacji zidentyfikowanych u pacjentów pozwoli w przyszłości na wprowadzenie terapii celowanej. Już w chwili obecnej, dane co do podłoża zdiagnozowanej u chorego encefalopatii padaczkowej są wskazówką stosowania lub niestosowania określonych leków przeciwpadaczkowych (tab. II).

## Encefalopatie padaczkowe – Diagnostyka molekularna

Diagnostyka zespołów padaczkowych, a w szczególności encefalopatii padaczkowych zawsze stanowiła duże wyzwanie ze względu na heterogenność genetyczną, ale także dużą zmienność fenotypową nawet przy tym samym podłożu genetycznym. Dla niektórych zespołów możliwe jest określenie korelacji genotyp-fenotyp, jednak w przypadku EIEEs jest to niezwykle trudne. Problemem jest tu zmienność fenotypowa zespołów, w tym ewolucja objawów klinicznych w czasie (zmiana typów napadów, wzór EEG czy ewolucja jednego zespołu w inny) i konieczność postawienia diagnozy jak najwcześniej. Zazwyczaj obraz kliniczny jest wtedy jeszcze niejednoznaczny, diagnoza może mieć wpływ na rodzaj stosowanej farmakoterapii (tab. II), czy postępowania z chorym (np. unikanie bodźców świetlnych u pacjentów z EIEE13, gen *SCN8A* czy kontrolowanie temperatury w przypadku EIEE6– zespołu Dravet, gen *SCN1A*).

**Tabela II:** Zespoły padaczkowe genetycznie uwarunkowane, w przypadku których jednoznaczna diagnoza molekularna – ustalenie etiologii choroby, ma wpływ na stosowaną terapię. Zaznaczono funkcje komórkowe produktów białkowych poszczególnych genów, wg. Poduri 2017 [42]. Table II. Genetic epilepsy syndromes in which unambiguous molecular diagnosis (etilology of the disease identification) influences treatment. Different cellular functions of the proteins encoded by the individual genes are depicted, according to Poduri 2017 [42].

Gen *(Gene)*	Postępowanie terapeutyczne *(Therapeutic action)*
Napięciowozależne kanały sodowe *Voltage-gated sodium channels*	
*SCN1A*	eliminacja fenytoiny i lamotriginy phenytoin and lamotrigine elimination
*SCN2A*	duże dawki fenytoiny (mogą być skuteczne) phenytoin high-dose (can be effective)
*SCN8A*	duże dawki fenytoiny (mogą być skuteczne) phenytoin high-dose (can be effective)
Napięciowozalezne kanały potasowe Voltage-gated potassium channels	
*KCNQ2*	do rozważenia retygabina w przypadkach mutacji utraty funkcji retigabine may be considered in cases of loss-of-function mutations
*KCNT1*	do rozważenia chinidyna w przypadku mutacji zmiany funkcji* quinidine may be considered in cases of gain-of-function mutations*
Zależne od ligandu kanały jonowe Ligand-gated ion channels	
*GRIN2A*	do rozważenia memantyna, w przypadku mutacji zmiany funkcji dekstrometorfan* memantine may be considered, in cases of gain-of-function mutations dextromethorphan
Transport glukozy Glucose transport	
*SLC2A1*	dieta ketogenna ketogenic diet
Metabolizm pirydoksyny Pyridoxine metabolism	
*ALDH7A1*	pirydoksyna pyridoxine
*PNPO*	*fosforan 5-pirydoksalu* pyridoxal-5-phosphate
Przewodzenie sygnału w neuronach Signal conduction in neurons	
*PRRT2*	karbamazepina carbamazepine
PLCB1	inozytol inositol
Cykl komórkowy Cell cycle	
TSC	do rozważenia ewerolimus everolimus may be considered

* potrzebne badania kliniczne (*clinical trials are needed*)

Wybór schematu diagnostyki pacjenta z zastosowaniem analizy DNA musi uwzględniać dane kliniczne, adekwatność proponowanego testu zarówno pod względem wykrywalności mutacji w kontekście badanego fenotypu jak i ograniczeń technicznych proponowanej metody. W przypadku padaczek/encefalopatii padaczkowych o wyborze postępowania diagnostycznego decydują wiek zachorowania i przebieg kliniczny choroby pacjenta. Najliczniejszą grupę kierowaną na badania stanowią chorzy z tzw. *„zespołami padaczkowymi plus”*, u których napadom mogą towarzyszyć cechy dysmorfii, niepełnosprawność intelektualna, autyzm i zaburzenia rozwojowe. Rekomendowanym badaniem jest tu wykonania tzw. kariotypu molekularnego, czyli analizy niezrównoważeń genomu z zastosowaniem hybrydyzacji porównawczej do mikromacierzy (*ang*. Chromosomal Microarray, CMA). Wykrywalność mutacji patogennych dla tej metody wynosi około 4-5% [37, 16]. Jeżeli zdiagnozowany u pacjenta zespół powodowany jest mutacjami w konkretnym genie/genach jak np. zespół Dravet (gen *SCN1A*) czy zespół Retta (gen *MECP2*) w pierwszej kolejności konieczna jest dokładna analiza tych genów – sekwencjonowanie z pełnym pokryciem wszystkich eksonów oraz identyfikacja rearanżacji wewnątrz genowych (delecji/duplikacji eksonów), co wymaga zastosowania metod o wyższej rozdzielczości niż CMA (np. MLPA). Alternatywną metodą jest zastosowanie sekwencjonowania panelowego, czyli równoległa analiza szeregu genów metodą NGS. Wykrywalność mutacji przy zastosowaniu sekwencjonowania panelowego szacuje średnio się na 20%, w przypadku pacjentów o wczesnym wieku zachorowania wartość ta jest wyższa i wynosi do 40% [38]. Panele „genów padaczkowych” mogą obejmować od kilkunastu do ponad 1000 genów. Zestaw badanych genów często związany jest z ukierunkowaniem badania np. pod kątem fenotypu lub wieku zachorowania. Coraz częściej w badaniach panelowych NGS możliwa jest analiza nie tylko wariantów sekwencyjnych, ale także delecji/duplikacji, co jest istotne w przypadku szeregu genów wrażliwych na dawkę jak *SCN1A, MECP2, SLC2A* czy *STXBP1*. Ograniczeniem badań celowanych jest fakt, że panele obejmują tylko te geny, które w danym momencie są znane. [39]. Logicznie, następnym krokiem w diagnostyce jest więc zastosowanie sekwencjonowania eksomowego (analiza wszystkich eksonów – sekwencji kodujących genomu). Przy zastosowaniu tej metody wykrywalność mutacji patogennych u pacjentów z padaczką wynosi około 35%, przy wyższych wartościach dla zespołów o wczesnym wieku zachorowania, szczególnie jeżeli chodzi o encefalopatie padaczkowe okresu noworodkowego [40]. Są to wartości porównywalne do analiz metodą sekwencjonowania panelowego. WES ma jednak podstawową zaletę, uzyskiwane dane, nawet jeżeli w danym momencie nie dają jednoznacznego rozpoznania, mogą zostać poddane reanalizie (obecnie sugeruje się nie częściej niż po 12 miesiącach) co w miarę poszerzania wiedzy na temat podłoża molekularnego zespołów padaczkowych pozwoli na identyfikację przyczyny choroby badanego pacjenta.

**Ryc. 3:**
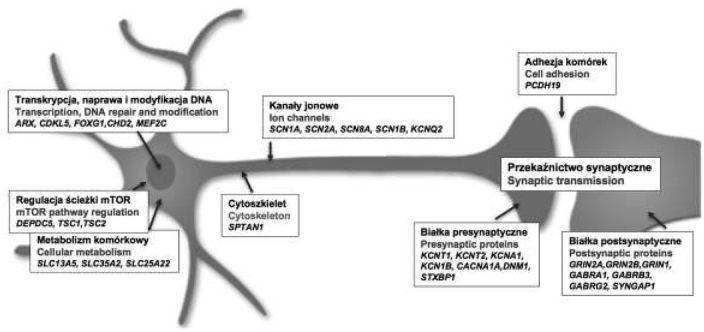
Geny i mechanizmy w komórkach neuronalnych zaangażowane w patofizjologię encefalopatii padaczkowych. Fig. 3. Genes and mechanisms (neuronal cells) involved in the pathophysiology of early infantile epileptic encephalopathies.

## Podsumowanie

Postęp technologiczny sekwencjonowania materiału genetycznego, który nastąpił w ostatnim czasie spowodował nagłe zmiany w genetyce padaczek, głównie poprzez gwałtowny wzrost zidentyfikowanych liczby genów, których mutacje stanowią podłoże chorób z tej grupy, a szczególnie encefalopatii padaczkowych. Możliwe stało się wprowadzenie testów diagnostycznych z wykorzystaniem sekwencjonowania eksomowego i panelowego. Skuteczność diagnostyczna tych testów oceniana jest na około 30% (dla EIEEs do 40%). Pomimo że testy genetyczne pozwalają w chwili obecnej na postawienie diagnozy molekularnej w szeregu przypadkach, to ich wyniki ciągle skłaniają do stawiania większej liczby pytań niż dają odpowiedzi. Systematycznie jednak poszerzają naszą wiedzę na temat etiopatofizjologii zespołów padaczkowych.

Czy postawienie diagnozy molekularnej u pacjenta, czyli poznanie podłoża molekularnego choroby, będzie miało dla tego pacjenta znaczenie, jeżeli chodzi o terapię, pytali w 2008 Delgado-Escueta i Bourgeois [3]. Miejmy nadzieję, że tak, i że wyniki obecnych badań nad genetyką i patofizjologią EIEEs umożliwią opracowanie nowych strategii terapeutycznych dla tych, niejednokrotnie określnych jako „*katastroficzne”* zespołów [41]. Jako że encefalopatie padaczkowe uważane są za modelowe, jeżeli chodzi o padaczki, opracowanie metod terapii dla tych zespołów, może doprowadzić do opracowania nowych metod farmakoterapii dla chorych z częstymi postaciami tej choroby.
